# Temperature-Dependent Electrical Transport and Microwave Network Characteristics of Rare-Earth (Y, Yb)-Coated Ge Thin Films

**DOI:** 10.3390/s26206609

**Published:** 2026-10-19

**Authors:** Mashael M. Altaiary, A. F. Qasrawi, Seham R. Alharbi

**Affiliations:** 1 Department of Physical Sciences, Faculty of Science, University of Jeddah, Jeddah 23442, Saudi Arabia; sralharbi@uj.edu.sa; 2 Department of Physics, Arab American University, Jenin P298, Palestine; atef.qasrawi@aaup.edu or atef.qasrawi@istinye.edu.tr; 3 Department of Electrical and Electronics Engineering, Istinye University, 34010 Istanbul, Turkey

**Keywords:** amorphous Ge, rare-earth coating, negative conductance, negative capacitance, two-port microwave network

## Abstract

Amorphous Ge thin films coated with rare-earth yttrium (Y) and ytterbium (Yb) nanosheets were investigated as multifunctional materials for electronic and microwave applications. Electrical characterization of as-grown Ge and coated Ge (Y, Yb) structures over 30–300 K and 0.01–6.0 GHz showed that Y and Yb coatings enhanced the low-temperature electrical conductivity by factors of 23.7 and 11.3, respectively. Charge transport was governed by thermally activated conduction and variable range hopping above about 120 K, while tunneling dominated in the 20–120 K range, indicating that rare-earth coating strongly modifies carrier localization and interfacial electronic states in Ge. In the 0.001–1.8 GHz range, Ge/Y and Ge/Yb also exhibited negative conductance and negative capacitance, supporting their potential for signal processing and sensing technologies. In parallel, sub-6 GHz two-port microwave measurements showed that the Y-coated sample exhibited the broadest common matched frequency range of 2.01–3.90 GHz and a balanced two-port response near 2.40 GHz. For Ge/Y, the minimum reflection coefficients were *S*_11_ = −37.84 dB at 2.61 GHz and *S*_22_ = −38.06 dB at 2.34 GHz, while *S*_21_ and *S*_12_ reached −24.12 dB and −23.96 dB, respectively, at 0.21 GHz. These results establish rare-earth coating as an effective approach for tuning the electrical and microwave network response of amorphous Ge thin-film structures and motivate their further investigation for sub-6 GHz microwave and wireless-device applications.

## 1. Introduction

Amorphous germanium thin films are of interest due to their vital role in electronics, thermoelectrics and optoelectronics. Germanium thin films as photoactive layer in metal-insulator-semiconductor devices exhibited highly photosensitive diode characteristics with rectification ratios exceeding 10^3^ and 100-fold increase in the photocurrent [1]. As a thermoelectric active layer Ge thin films coated onto Ag substrates exhibited maximum electrical conductivity, Seebeck coefficient and thermo-power factor of 9.22 (Ωcm)^−1^, 366.39 μVK^−1^ and 123.87 μW m^−1^K^−2^, respectively, demonstrating the novelty of the films as thermoelectric power generators [2]. In addition, germanium thin films deposited onto flexible Kapton tape were used as gama ray monitors capable of monitoring doses up to a level of 12 kGY [3]. Moreover, germanium layers as a high hole mobility and “non-localized states” amorphous layer are regarded as an exceptional amorphous layer exhibiting free carrier concentration of the order of 10^18^ cm^−3^ and carrier mobility of 100 cm^2^/VS, making it promising substrate for thin-film transistor technology [4].

Germanium thin films are very attractive in terms of their functionality in various technologies. However, germanium thin films face a challenge when used in electronic devices. This is because germanium has an inherently unstable surface due to its tendency to form a native oxide layer. This has always hindered its smooth incorporation in electronic devices [5]. Formation of the chemically unstable GeO or GeO_2_ on the top surface of the films degrades the surface quality of the material. To avoid the high electrical resistivity resulting from surface oxidation, here in this work, we are motivated to introduce metal nanosheets on the surface of Ge films so that electrical conduction is significantly improved. For this purpose, two 100 nm thick rare-earth metal nanosheets (Y, Yb) were deposited onto the surface of Ge. Ytterbium is selected because of its high transparency to ultraviolet–visible–infrared light spectrum [6]. It is reported as an ideal metal for interface engineering, passivation and contact property control [7]. Yttrium is also preferred to reduce the defects existing on the surface of amorphous germanium. Yttrium is known as a getter material of the traps [8]. These rare-earth nanosheets can reduce the surface oxidation, interface defects and trap states. These negative effects which exist in amorphous materials like germanium degrade carrier transport and device stability. Hence, using Y is expected to enhance the interface quality, electrical reliability and long-term performance of the devices [9].

In spite of these benefits, the main problem still needs to be solved of whether or not the rare-earth surface modification of amorphous Ge allows one to simultaneously control its carrier transport at low temperatures, interfacial relaxation and high-frequency microwave electrical properties. In particular, the relationship between the effect of coatings on localized states and the carrier transport that determines the impedance and scattering properties of the structure at high frequencies has not been sufficiently studied. This problem is crucial, as the microwave properties of the thin-film structure not only depend on its geometrical parameters, but also on its frequency-dependent conductivity, interfacial polarization, trapping kinetics and impedance.

Therefore, the goal of the current study is to explore the possibility of using Y and Yb nanosheets for tailoring the electronic properties of amorphous Ge via altering its localized states and interfaces. In particular, the impact of the surface coating on the temperature-dependent conductivity and identification of the main conduction mechanisms (thermally activated conduction, variable range hopping, and low temperature tunneling) is determined first. Then, the connection between the material properties of the investigated system and complex admittance, negative capacitance/negative conductance effect, and complex impedance properties of the Ag/Ge/(Y,Yb)/Ag structure is established. Finally, the effect of the identical surface modification on the two-port microwave scattering response of the sample in the sub-6 GHz range is evaluated. Using identical device geometry with different Ge surface coatings, it is intended to identify whether the rare-earth-induced alteration of electronic and interface properties leads to specific changes in microwave resonance, reflection, and inter-port transmission. In this way, the work aims to establish a physical link between nanoscale interface engineering and the tunability of Ge-based microwave thin-film structures.

Previous studies [10,11] have demonstrated that the microwave response of thin-film structures can be influenced by material conductivity, dielectric properties, interfacial polarization, and device geometry. However, the influence of rare-earth Y and Yb surface coatings on the combined electrical transport and two-port microwave network characteristics of amorphous Ge remains insufficiently explored. The novelty of the present work lies in demonstrating experimentally that Y and Yb coatings produce distinct changes in carrier transport, interfacial relaxation, complex impedance, and microwave scattering characteristics under identical nominal geometrical conditions. Particularly, Y coating broadens the common impedance-matched frequency range, whereas uncoated Ge provides stronger inter-port isolation. These findings establish rare-earth surface modification as an experimental approach for tailoring Ge-based microwave thin-film networks without altering their nominal geometry.

## 2. Experimental Details

**Figure 1:**
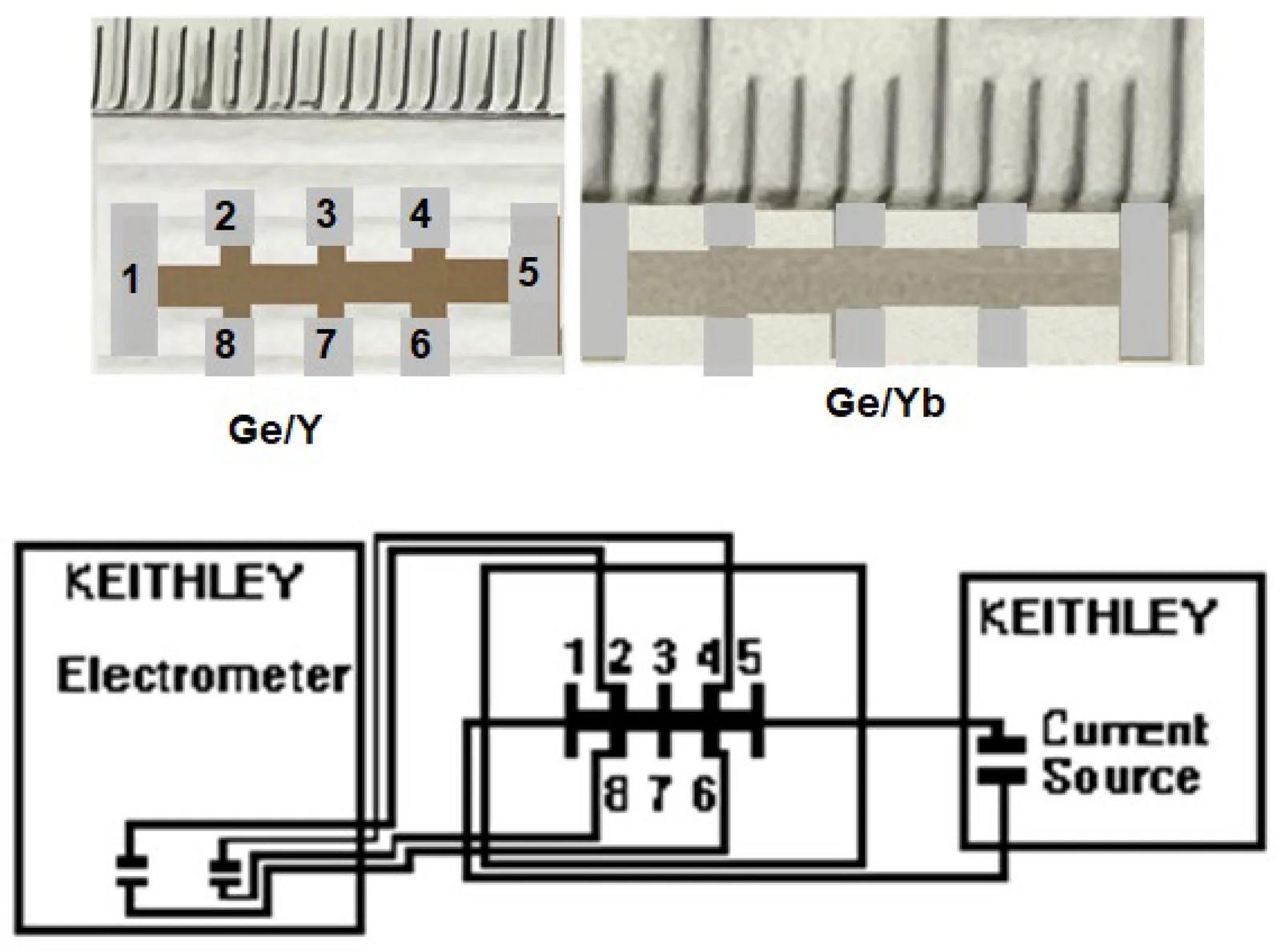
Optical images and schematic for the Hall bar geometry used in determining the electrical conductivity.

Germanium thin films are coated on glass and Ag thin-film substrates using the thermal evaporation technique under a vacuum pressure of 10^−5^ mbar. The evaporation source material was high-purity (99.99%) germanium crystal lumps ordered from Alfa Aesar firm, Ward Hill, MA, USA. Silver substrates are also prepared by the same method from Ag grains (Alfa Aesar) of 99.99% purity. The thicknesses of the Ge and Ag films were 500 nm, as monitored during deposition using an Inficon STM-2 thickness monitor (East Syracuse, New York, NY, USA) installed in the thermal evaporator (NORM-600 V2, NORM Electronics LTC., Athens, Greece). The produced Ge films were subsequently coated with 100 nm thick Y or Yb layers. The SEM measurements were performed to characterize the surface morphology of the films; therefore, the reported layer thicknesses refer to the values monitored during thermal evaporation and were not extracted from cross-sectional SEM measurements. The produced films are then coated with 100 nm thick yttrium (Alfa Aesar, 99.99%) and ytterbium (Alfa Aesar, 99.99%) nanosheets. The coating of the metal nanosheets were actualized in a NORM-600, thermal evaporator under a vacuum pressure of 10^−5^ mbar. The sources were Yb (99.99%, Alfa Aesar) and Y (99.99%, Alfa Aesar) crystal lumps. The respective evaporation rates were 12 Å/min and 15 Å/min. Both uncoated and rare-earth metal-coated films is subjected to X-ray, scanning electron microscopy, impedance spectroscopy and temperature-dependent electrical measurements using Rigaku Miniflex 600 XRD unit (Rigaku corp., Tokyo, Japan), COXEM-200 SEM unit (COXEM Co. LTd., Daejeon, Republic of Korea), Agilent 4291B impedance analyzer (Agilent Technologies, Santa Clara, CA, USA) equipped with dielectric test fixture and a 10 K- ARS closed cycle helium cryostat (Advanced Research Systems, Inc. (ARS), Macungie, PA, USA) attached to Keithley current-voltage system, respectively. The electrical conductivity (*σ* = *L*/(*Rwd*)) was measured using a Hall bar samples with length *L* = 1.2 cm, *w* = 0.2 cm and thickness (*d*) of 500 nm for Ge and 600 nm for others. The optical images and schematic for the Hall bar samples is shown in Figure 1. The measurement setup is also illustrated in Figure 1. A constant current was applied through the sample (*I*_15_ in the circuit diagram) using a Keithley programmable current source, which provides currents ranging from 0.1 nA to 100 mA with a compliance voltage of up to 100 V. The voltage drops across contacts 2 and 4 (V_24_) and contacts 6 and 8 (V_68_) were measured using a Keithley electrometer with a voltage sensitivity of 10 mV. The applied current, I, was plotted separately as a function of V_24_ and V_68_. The slopes of the two I–V characteristics were determined, and their average value was used to calculate the electrical conductivity of each sample. The dominant n-conduction type was explored using the hot probe technique. The frequency-dependent impedance and admittance measurements were performed over 0.001–1.80 GHz using an Agilent 4291B impedance analyzer, whereas the two-port scattering parameters were measured over 0.01–6.0 GHz using a Rohde & Schwarz vector network analyzer (Rohde & Schwarz GmbH & Co. KG, Munich, Germany). For the two-port measurements, two identical circular Ag top contacts, each having an area of 0.0314 cm^2^, were separated by 10 mm, while the lower Ag film served as the common ground. The two circular contacts were connected sequentially as ports 1 and 2, and the complete scattering matrix (*S*_11_, *S*_22_, *S*_21_, *S*_12_) was measured. The same contact geometry and measurement configuration were maintained for the Ge, Ge/Y, and Ge/Yb structures so that changes in the measured scattering response could be compared under identical geometrical conditions. The dimensions of contact and the spacing have been maintained constant as a part of the experiment and have not been determined on the basis of electromagnetic optimization. It follows that this comparison aims at measuring the effect of material change in similar geometrical conditions rather than optimization of geometry of microwaves/antennas. The reproducibility of the fabrication process was examined through three independent deposition runs under identical nominal conditions. Three devices of each structure (Ge, Ge/Y, and Ge/Yb) were measured in each run, giving nine measured devices per structure and 27 devices in total. The independently fabricated devices exhibited reproducible electrical and microwave characteristics. The estimated overall uncertainty ranged from 2% to 10%, accounting for both device-to-device variability and measurement instrument uncertainty.

## 3. Results and Discussion

### 3.1. Structural and Morphological Analyses

**Figure 2:**
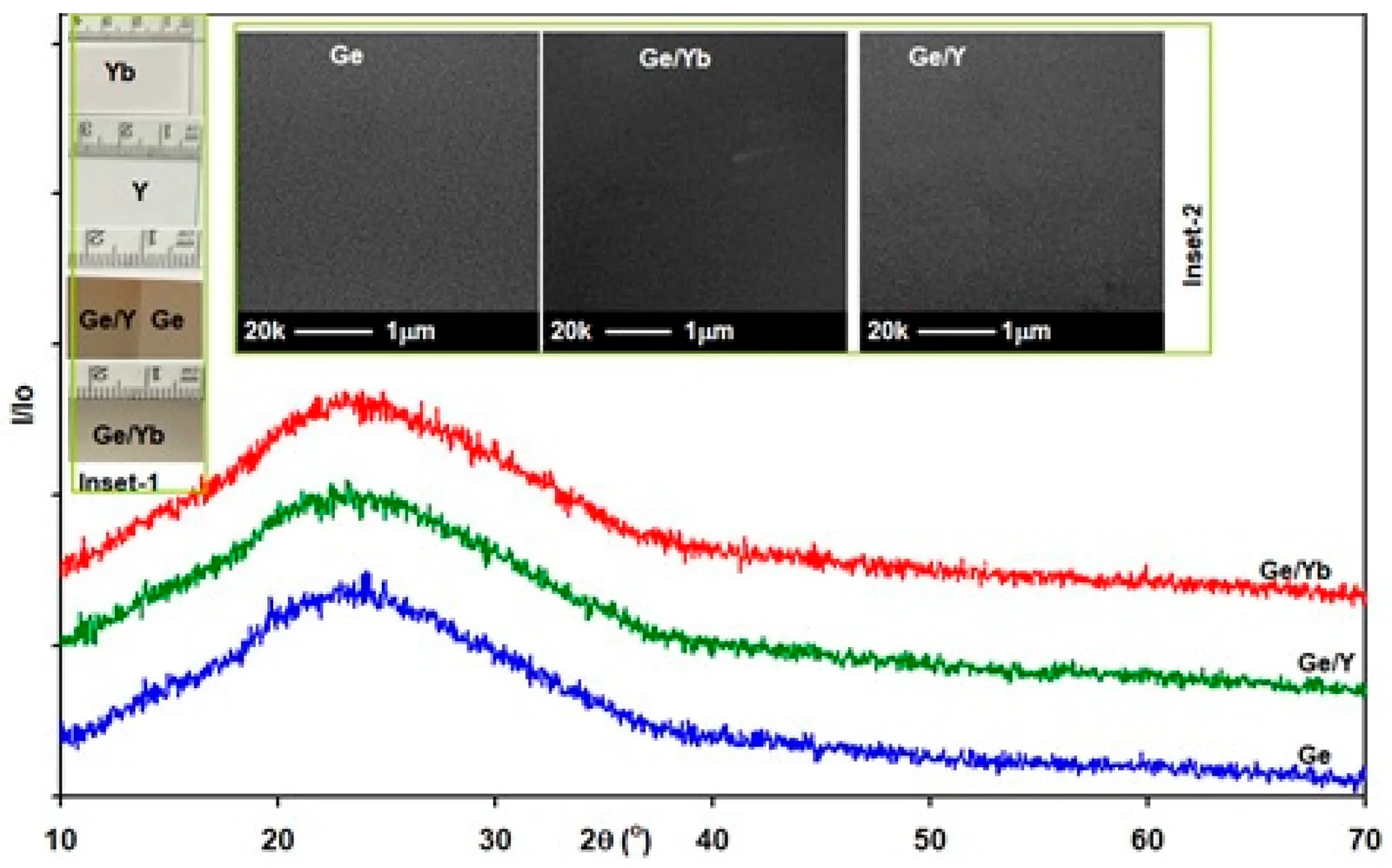
The X-ray diffraction patterns for amorphous Ge films coated with rare-earth Y and Yb metal nanosheets. Inset-1 showing the optical images of Y, Yb, Ge, Ge/Y and Ge/Yb films. Inset-2 of the figure showing the scanning electron microscopy measurements for the studied films.

The optical images for the rare-earth metal nanosheets coated onto glass and Ge films are shown in inset-1 of Figure 2. The transparency of Y and Yb nanosheets is clearly observed from the off-white color of the coated films. While yttrium converted the color of Ge from bright brown to dark brown, Yb converted the color to bright grey. Inset-2 of Figure 2 also demonstrates the scanning electron microscopy (SEM) images for Ge thin films and Ge coated with Y and Yb. None of the images demonstrated any grains or clusters. The films are very homogeneous showing the same surface morphology. The SEM images reveal smooth and homogeneous surfaces without clearly resolved grains or clusters, while the absence of sharp diffraction peaks in the XRD patterns confirms the predominantly amorphous nature of the films. On the other hand, Figure 2 illustrates the X-ray diffraction patterns for the films under study. No sharp peaks are observed in the XRD patterns confirming the amorphous nature of growth of the films. The rare-earth nanosheets coating did not alter the surface morphology or crystal structures of Ge thin films. The amorphous nature of growth of Ge films is usually accompanied with high defect concentration. High defects are responsible for scattering of charge carriers leading to higher electrical resistivity values [5].

### 3.2. Temperature-Dependent Electrical Conductivity and Transport Mechanisms

**Figure 3:**
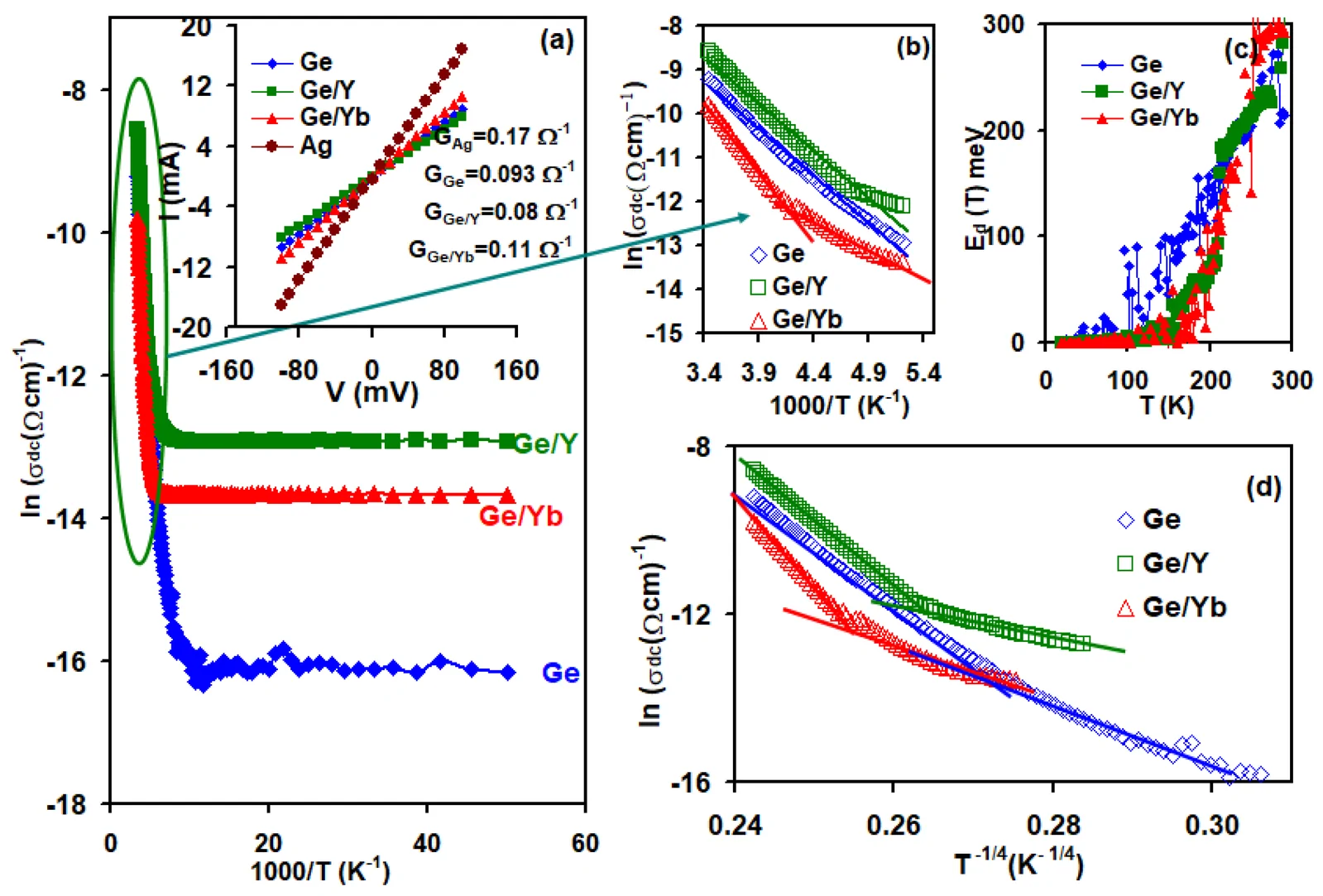
(**a**) The Arrhenius plots of electrical conductivity in the 20–300 K temperature range and (**b**) the Arrhenius plots of electrical conductivity in the high temperature range, (**c**) showing the temperature dependence of donor energy level on temperature, and (**d**) the $\ln\left(\sigma \right)-T^{-\frac{1}{4}}\;$ variations in the low-temperature range showing the domination of variable range hopping transport in the films. Inset of (**a**) showing the current (I)-voltage (V) characteristic curves for the investigated device channels.

The room-temperature current–voltage (*I–V*) characteristics of the studied samples are shown in the inset of Figure 3a. All samples exhibit nearly linear *I* − *V* behavior over the investigated voltage range, indicating Ohmic conduction and good electrical contact between the electrodes and the samples. The corresponding *I* − *V* slopes (*G* = *dI*/*dV*) are 0.17 Ω^−1^, 0.093 Ω^−1^, 0.08 Ω^−1^ and 0.11 Ω^−1^ for Ag, Ge, Ge/Y and Ge/Yb, respectively. It should be emphasized that linearity of the *I* − *V* curves does not imply identical electrical conductivities. According to Ohm’s law, the slope of each *I* − *V* curve (*I*/*V*) represents the conductance, which is related to the conductivity through the sample geometrical dimensions. Therefore, the different slopes observed for Ge, Ge/Y, and Ge/Yb are consistent with their different conductivity values presented in Figure 3a. In particular, the changes in slope upon Y and Yb incorporation reflect the corresponding modification of charge transport in the Ge-based samples.

The temperature-dependent electrical conductivities (*σ_dc_*) of the Ge, Ge/Y and Ge/Yb films are studied in the temperature range of 20–300 K. The respective values of the electrical conductivity at room temperature are 9.97 ×10^−5^ (Ωcm)^−1^, 1.92 ×10^−4^ (Ωcm)^−1^ and 5.53 ×10^−5^ (Ωcm)^−1^. The numerical values here indicate that Y nanosheets coating increased the electrical conductivity by 1.9 times whilst Yb decreased it by 1.8 times. The *σ_dc_* − *T*^−1^ variations shown in Figure 3a show that the decay of *σ_dc_* with *T* is faster for uncoated films compared to those coated ones. The electrical conductivity of Ge thin films decreased from 9.97 ×10^−5^ (Ωcm)^−1^ to 6.33×10^−7^ (Ωcm)^−1^ as the temperature decreased from 290 K to 160 K and continued decreasing, reaching a value of 8.09 ×10^−8^ (Ωcm)^−1^ at 85 K. In the temperature range of 20–80 K, *σ_dc_* of uncoated Ge tends to be temperature invariant. This behavior of electrical conductivity illustrated by thermal activation of conductivity over a wide range of temperature terminates at higher temperatures for Y- and Yb-coated films. Namely, *σ_dc_* tends to be temperature invariant in the temperature range of 20–120 K and 20–140 K for Ge/Y and Ge/Yb, films, respectively. The average conductivity value in the low-temperature regions (20–90 K) is 1.02 × 10^−7^ (Ωcm)^−1^, 2.44 × 10^−6^ (Ωcm)^−1^ and 1.16 ×10^−6^ (Ωcm)^−1^ for Ge, Ge/Y, and Ge/Yb, respectively. Yttrium and ytterbium nanosheets coatings enhanced the low temperature electrical conductivity by 23.7 and 11.3 times, respectively. On the other hand, the Arrhenius plots of the electrical conductivity which are presented in Figure 3b showed linear variations consistent with the thermal excitation of thermally activated charge carriers following the equation [12].

*σ_dc_* = *σ_o_* exp(−*E_d_*/*kT*). (1)

Here *σ_o_* is the pre-exponential factor and *E_d_* is the donor level which conduction electrons originated from. As seen from Figure 3b, the linear fittings of the ln(*σ*) − *T*^−1^ variations shown by solid lines reveal conductivity activation energies that are assumed to be related to donor levels. *E_d_* values of 184 ± 5 meV and 209 ± 5 meV are, respectively, determined for Ge and Ge/Y in the temperature range of 200–300 K and 215–300 K. For Ge/Yb films, in the same temperature range, two donor levels of values of 291 meV and 138 meV existed above and below 250 K, respectively.

In an attempt to understand whether the donor levels are discrete or just a continuum band, the derivative of Equation (1) was executed and the temperature dependence of the donor levels was extracted. In accordance with this approach, *E_d_*(*T*) takes the form [12].

(2) $$E_{d}\left(T\right)=-k\frac{d(\ln(\sigma )}{d(1/T)}$$

The donor level measured from the conduction band edge (*E_c_*(*T*)) as function of temperature is shown in Figure 3c. It is clear from the figure that for all the samples under study, *E_d_*(*T*) is a strong function of *T* and sharply decreases with decreasing *T*, reaching very low values at low temperature. The lowering of the temperature forces the impurity levels to be shallower. However, this behavior seems to be in conflict with the standard behavior of semiconductors. The general behavior of semiconductors are that raising temperature activates shallower donor levels. This usually happens because the donor binding energy is function of dielectric constant (dielectric screening) and effective mass ($E_{d}\propto \frac{m^{*}}{\varepsilon^{2}}$). The increase in the temperature is accompanied with changes in the screening which reduces the donor binding energy shifting the donor levels closer to the conduction band edge [13]. This trend of variation opposes what is shown in Figure 3c, indicating the domination of other additional conduction mechanism in the samples like defect restructuring, compensation and charge carrier localization with varying temperature [14].

As a sharing transport mechanism, we considered the possible domination of variable range hopping mechanism (VRH) in the films. In accordance with this approach, the electrical conductivity is given by the relation [12,15].

(3) $$\sigma_{\mathrm{dc}}=\sigma_{o1}\exp\left[-{\left(\frac{T_{o}}{T}\right)}^{1/4}\right]$$

*σ_o_*_1_ = *e*^2^*a*^2^*V_ph_N*(*E_F_*) (4)

(5) $$T_{o}=\frac{18\gamma^{3}}{\mathrm{kN}(E_{F})}$$

According to Mott and Davis [15], *a* represents the hopping distance between sites, *ν*_ph_ ~ 10^13^ s^−1^ is the phonon frequency, the decay constant *γ* = 1/*ξ* with *ξ* being the localization length and *N*(*E_F_*) is the density of localized states near the Fermi level. Based on the results of Equations (3)–(5), the average hopping range, *R*, and the average hopping energy, *W*, are calculated using the relations [8,15].

(6) $$R={\left[\frac{9}{8\mathrm{\pi \gamma kTN}(E_{F})}\right]}^{1/4}$$

(7) $$W=\frac{3}{4\pi R^{3}N(E_{F})}$$

The fitting of the electrical conductivity as function of *T*^−1/4^ which is shown in Figure 3d reveal two slopes of variations for each sample. The temperature ranges where the VRH mechanism is valid are listed in Table 1 together with the estimated VRH parameters. A localization length of *ξ* = 10 Å was assumed for all the samples. The average hopping distance was evaluated at 250 K for the high temperature range (above 200 K) and at 160 K for the low-temperature range (below 200 K). The validity of this transport mechanism is assured by the values *W* > *kT*, *γR*≫1 and *T*_0_ > 10^3^ K [15]. The tabulated data indicate that all *γR*≫3 and *W*≫*kT* while *T_o_* >> 10^5^ K indicating the possible domination of VRH in the studied films. As a valid rule for each sample estimated from Table 1, the higher the temperature range, the higher the degree of disorder (*T_o_*), the less the density of localized states near Fermi level, the larger the hopping range and the higher the average hopping energy. At high temperature where the donor levels are strongly sharing in the transport mechanism, the density of localized states near Fermi levels decreased upon coating the film with Y and Yb, whilst it significantly increased after coating in the lower-temperature range where *E_d_* levels are less contributive. The lower temperature range (below 200 K) indicates the stronger probability of variable range hopping, in that range Y and Yb coatings decreased the degree of disorder from 4.91 × 10^7^ K to 2.66 ×10^6^ K and 1.036 ×10^6^ K, respectively. They also increased *N*(*E_F_*) by one and two orders of magnitude, respectively.

Physically, the differences between the two hopping ranges come from changes in the dominant hopping environment with temperature. At higher temperatures, carriers can access a wider range of localized states and hop through deeper and more disordered regions. This process need more energy and longer paths. At lower temperatures, transport is limited to the states nearest to the Fermi level. After coating with Y or especially Yb, these nearby states become well connected through improved interfacial overlap and screening. As a result, hopping becomes easier, shorter, and less energetic [15]. In addition, from a localization perspective, Ge/Yb is expected to show stronger electronic localization than Ge/Y. This is because Yb has highly localized 4*f* states, while Y mainly provides more extended 4*d*/5*s*-type states. The localized states from Yb trap carriers and form narrow defect-like states near the interface. In contrast, Y tends to mix better with Ge and allows for improved wavefunction overlap, resulting in a larger localization length and easier hopping. In summary, Ge-Yb promotes stronger localization, whereas Ge-Y supports stronger delocalization and interfacial coupling [16].

In the lowest temperature range (less than 120 K), the electrical conductivity for all studied samples is temperature invariant indicating that the thermal activation energy is suppressed and electrical conduction is dominated by charged particle tunneling through interfacial barriers. Such behavior was described by the fluctuation-induced tunneling (FIT) equation [17].

(8) $$\sigma_{T}\left(T\right)=\sigma_{\mathrm{oT}}\exp(-\frac{T_{1}}{T+T_{01}})$$

where the tunneling barrier strength $T_{1}=\frac{8\mathrm{\varepsilon A}\phi_{o}^{2}}{ke^{2}\omega }$ with *A* being the effective area of the junction, *ω* and *ϕ_o_* is the barrier width and barrier height, respectively, and *T_o_*_1_ is a characteristic crossover temperature that reflects how strongly thermal fluctuations can assist tunneling at higher temperatures [17].

In accordance with this theory, at low temperature, the nearly invariant conductivity indicates that thermal activation becomes weak and charge transport is dominated by tunneling across interfacial barriers rather than over-barrier hopping. In the fluctuation-induced tunneling model mentioned above, *T*_1_ reflects the barrier strength and *T_o_*_1_ marks the temperature scale below which conductivity becomes weakly temperature-dependent; thus, when *T*≪*T_o_*_1_, the conductivity tends to a plateau-like behavior because carriers tunnel through barriers with little thermal assistance. This interpretation is consistent with Sheng’s FIT model for disordered systems [17].

### 3.3. Negative Conductance and Negative Capacitance Effects

**Figure 4:**
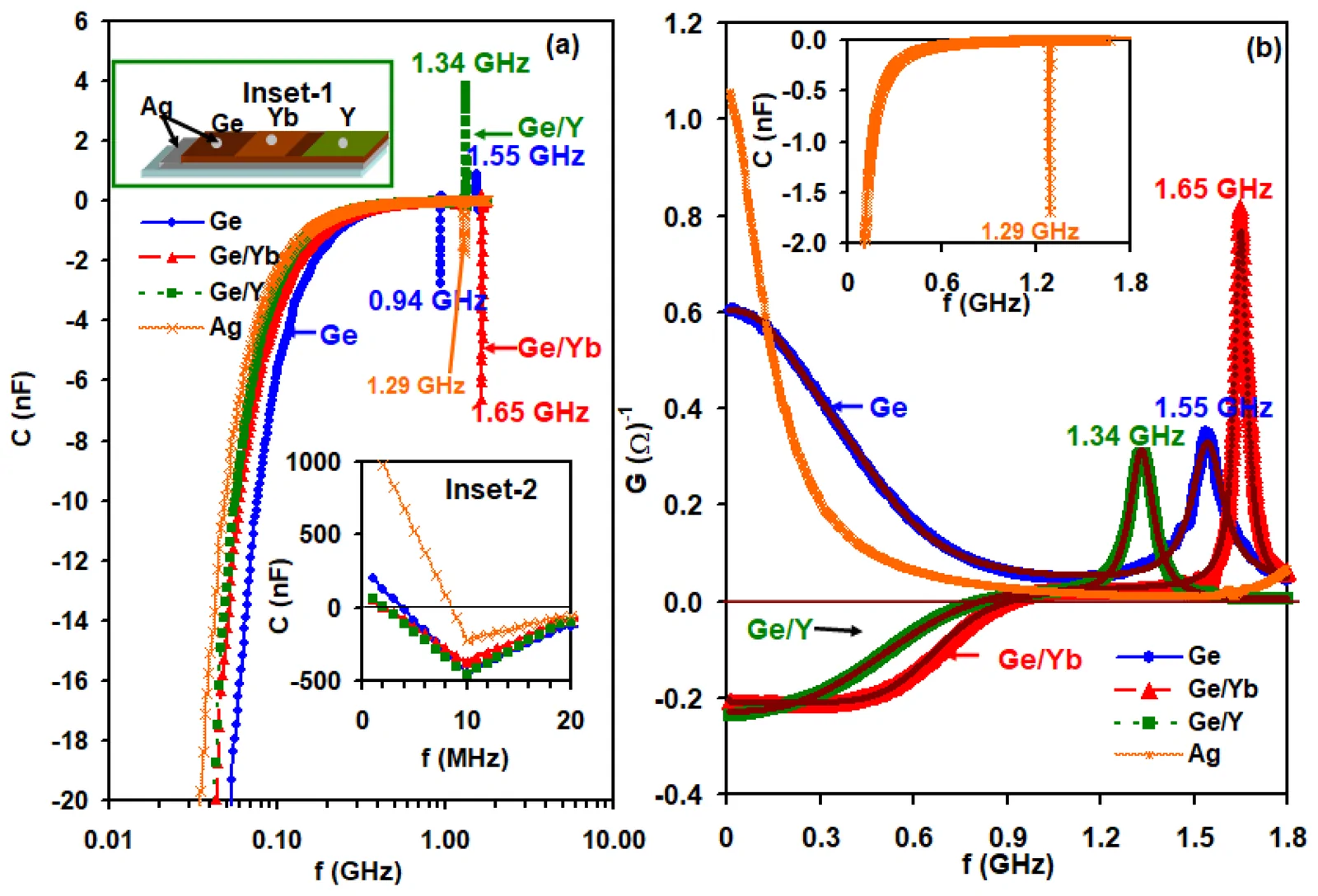
(**a**) The capacitance and (**b**) conductance spectra for glass/Ag and Ag/Ge (Yb, Y)/Ag microwave resonators. Inset-1 and inset-2 of (**a**) showing the schematics and the low-frequency measuring range of the capacitance spectra, respectively, for the rare-earth metal-coated device. Inset of (**b**) displaying the capacitance of Ag substrate alone.

As practical application, germanium thin films were deposited onto Ag thin-film substrate and partially coated with the rare-earth metal Y and Yb nanosheets. The top layers were then coated with Ag point contacts to form two terminal electronic devices. The schematic of the device which is composed of three channels (Ge, Ge/Y, Ge/Yb) is illustrated in inset-1 of Figure 4a. The frequency-dependent electrical measurements were performed using an Agilent 4291B impedance analyzer equipped with a dielectric test fixture. The three channels were sequentially imposed between the terminals of the impedance analyzer which generates waveform signals of the form of $\tilde{V}=V_{o}e^{\pm \mathrm{i\omega t}},\;(\omega =2\mathrm{\pi f})$. The frequency (*f*) domain was varied in the range 0.001–1.80 GHz and the amplitude was kept low (*V_o_* < 0.1 V) to allow detection of weak signals. At each frequency, the complex impedance *Z*(*ω*) was measured and converted to the complex admittance $Y\left(\omega \right)=\frac{1}{Z\left(\omega \right)}=G\left(\omega \right)+\mathrm{iB}(\omega )$. The conductance was obtained from the real component, *G*(*ω*) = *Re*{*Y*(*ω*)}, while the capacitance was determined independently from the imaginary component as $C\left(\omega \right)=\frac{\mathrm{IM}\left[Y\left(\omega \right)\right]}{\omega }$. Therefore, variations in resistance or conductance with frequency are inherently accounted for in the complex impedance measurement and do not prevent extraction of the capacitance. Consequently, it can be deduced that a negative value for the extracted capacitance will lead to a negative imaginary part of the admittance for the device in question, and thus will indicate frequency-dependent reactivity of the device rather than static geometrical capacitance. The measured capacitance spectra are illustrated in Figure 4a. It can be seen from the figure that all four channels reveal negative capacitance effect (NC) including Ag. Negative capacitance behavior is found in the structures based on Ge as well, with a negative or positive resonant effect depending on the channel material. To estimate the role that the lower Ag electrode may play in producing the observed NC effect, measurements of the corresponding capacitance and conductance spectra of the Ag/glass film were conducted under identical experimental conditions and are presented in Figure 4a and inset of Figure 4b. One can observe that the Ag/glass control shows negative apparent capacitance in a significant fraction of the low-frequency range under study, together with an additional feature near 1.29 GHz. This means that the Ag electrode and the electrode interface response make their contribution to the reactive background detected in the full devices. However, the spectrum becomes clearly modified when the Ge and Y or Yb films are deposited on top of the Ag electrode, and clear resonant features become observable near 0.94 and 1.55 GHz for Ge, 1.34 GHz for Ge/Y, and 1.65 GHz for Ge/Yb. Thus, the NC response of the full device structure is not due to the Ag film alone; it reflects the collective frequency-dependent response of the Ag electrode, Ge layer, and Y/Yb interfaces. The capacitance spectra of Ge channel are mostly negative and exhibited negative resonance at 0.94 GHz followed by positive resonance at 1.55 GHz. Coating this channel with yttrium nanosheets forced disappearance of the negative resonance in Ge channel and the positive resonance peaks appeared which appeared at 1.55 GHz remained positive and shifted to 1.34 GHz. On the other hand, coating Ge channels with Yb nanosheets forced disappearance of the negative and positive resonance peaks and new negative resonance peak appeared at 1.65 GHz.

It should be noted that the lower Ag film acts as the common electrode and forms a Ag/Ge interface; therefore, it participates in the interfacial charge redistribution and polarization contributing to the measured complex admittance. The measured resonance consequently represents the collective response of the complete Ag/Ge/Yb/Ag structure rather than that of the Yb layer alone. Since the lower Ag/Ge interface is common to all three devices, however, the emergence of the 1.65 GHz resonance upon Yb coating is more reasonably associated with modification of the overall interfacial relaxation and reactive response by the additional Ge/Yb interface, rather than being attributed solely to the lower Ag film.

NC effect is an important feature of electronic components playing significant role in reducing power consumption and enhancing switching performance in the devices. Inverting negative resonance into positive resonance achieved by Y coating at 1.34 GHz means switching within a period of time of 0.75 ns whilst Yb coating inverts the positive resonance observed at 1.55 GHz in Ge channels to negative resonance at 1.65 GHz. Switching is actualized within 0.61 ns. In addition NC effect is utilized in reducing the operating voltage of devices and enhancing charge control and voltage amplification [18], NC effect is also used in improving energy-efficient logic and memory devices in addition to their ability to support low power 5G/6G technology applications [19].

The negative capacitance behavior measured at microwave frequencies may be considered as a negative reactive element contribution to the admittance of the device. By incorporating properly such a response in a microwave circuit, this negative contribution could serve to cancel the capacitive contribution and hence change the overall input impedance. The application of this technique is pertinent to non-Foster impedance matching, where negative reactive components are used to tune the reactances and achieve impedance matching for microwave circuits and antennas. Negative-capacitance components could also be used in active filters and resonant microwave circuits. Hence, in the proposed devices, the observed negative capacitance may be useful in tuning the frequency dependence of impedance and resonance of Ge-based microwave devices. Negative capacitance results from the subtle equilibrium between the in-plane and out of plane polarization states in the vicinity of Bloch point (polarization changes abruptly in all three dimensions at this point) [20]. For our amorphous samples, this point is regarded as a localized singularity at which the polarization direction becomes undefined, separating different in-plane and out of plane polarization configurations. NC effect can also result from the minority carrier injection caused by the accumulation of minority carriers at the junction interfaces [21].

As a complementary issue, the capacitance which was measured down to the low-frequency range of 1–20 MHz is depicted in Inset-2 of Figure 4a. Here, the goal is showing the low-frequency behavior that is closer to the DC limit. At these frequencies, the structures display a notable frequency-dependent reduction of capacitance from positive values at low frequencies to negative ones at higher frequencies. While glass/Ag exhibits the highest low-frequency capacitance value, the coating of Ge with subsequent application of Y or Yb results in a significant change in the capacitive behavior. The transition from positive to negative capacitance signifies the effect of increasing frequency on the reactive response and electrode/interfacial polarization and charge relaxation mechanisms.

Figure 4b shows the conductance (*G*) spectra of the investigated devices. The Ag/glass control exhibits positive conductance throughout the investigated frequency range, although it shows an apparent negative-capacitance response, as illustrated in Figure 4a. This observation indicates that, although the lower Ag film contributes to the reactive response of the complete structures, it does not by itself account for the negative-conductance behavior observed in the Y- and Yb-coated Ge channels. The Ge channel also exhibits positive conductance, which initially decreases with increasing signal frequency over the range 0.001–1.34 GHz. At higher frequencies, *G* increases and reaches a maximum at 1.55 GHz, where both *C* and *G* are positive. In contrast, the Ge/Y and Ge/Yb channels exhibit negative conductance (NG) over the frequency ranges 0.001–0.84 GHz and 0.001–0.89 GHz, respectively. Within these frequency ranges, the magnitude of the negative conductance decreases with increasing frequency until *G* crosses zero and becomes positive at the corresponding critical frequencies. In the higher frequency domain where the conductance is positive the maximum conductance peak observed at 1.55 GHz shifts to 1.34 GHz and to 1.65 GHz as the Ge channel was coated with Y and Yb nanosheets. NG effect is significant property of thin-film transistors enabling oscillators, multivalued logic devices, memory elements and other nonlinear high speed electronics [22,23]. Considering the AC admittance effect [24,25],

(9) $$Y(\omega )=G(\omega )+i\omega C(\omega )=G_{\infty }+i\omega C_{\infty }+\sum_{k=1}^{n}\frac{\Delta G_{k}}{1+(\mathrm{i\omega}\tau_{k}{)}^{1-\alpha_{k}}}$$

with *G_∞_* and *C_∞_*, *τ_k_* and *α_k_* being the high-frequency conductance and geometric capacitance, the relaxation time and the distribution of the relaxation time, respectively. According to this equation, if one delayed channel is active or feedback driven (Δ*G_k_* < 0) then the real part of admittance (*G*(*ω*) = *Re*|*Y*(*ω*)| < 0) can be negative over a finite frequency domain. Here carrier injection and release feedback dominate the normal conductive response. Such effect appears when conductance is strongly dominated by trapping (defect) dynamics, interface states and non-equilibrium charge carrier exchange [24].

Mathematically, using the extended frequency-domain model with the relation [25],

(10) $$G\left(f\right)=c+\frac{a_{1}}{1+(f/f_{1}{)}^{m_{1}}}+\frac{a_{2}}{1+(f/f_{2}{)}^{m_{2}}}+\frac{A\gamma^{2}}{{\left(f-f_{o}\right)}^{2}+\gamma^{2}}$$

Here $\tau_{i}=\frac{1}{2\pi f_{i}}$, *ω* = 2*πf* and *m_i_* ≠ 2 because of NG effect, interfacial relaxation, trapping and distributed localized states leads to a non-Drude behavior. This is physically more realistic because it captures a distribution of relaxation processes rather than one single free-electron relaxation channel [25].

In the above equations, the relaxation times represent how fast charge carriers or dipolar/interfacial polarization can respond to the applied AC field. A short relaxation time means the carriers follow the field rapidly, which usually indicates easier charge redistribution, weaker trapping, and better electronic coupling across the channel or interface [26]. A long relaxation time means that the response is delayed, which points to stronger carrier localization, deeper trap states, or slower interfacial polarization processes [26]. The experimental data of the conductance spectra are reproduced using Equation (10) and presented as pink colored solid circles in Figure 4b. The fitting procedure resulted in the physical parameters listed in Table 2. The table indicates that Ge/Y exhibits shorter relaxation times than bare Ge, meaning that Y coating appears to facilitate faster carrier motion and quicker interfacial charge adjustment [27]. In contrast, Ge/Yb exhibits a very large τ_1_ value, which physically suggests that Yb introduces slow localized states or stronger trapping centers that retard the carrier response at low frequency. The similar *τ_o_* values for all samples imply that the high-frequency resonance process is nearly common to the three channels, while the major differences arise mainly from the low- and intermediate-frequency relaxation mechanisms. The larger peak amplitude *A* and smaller linewidth γ for Ge/Yb also indicate a stronger and sharper high-frequency resonance contribution. In addition, the resonance frequencies around *f_o_* = 1.3–1.7 GHz (Table 2) for all of the samples indicate a common high-frequency polarization or carrier oscillation process, but the smaller linewidth (*γ*) for Ge and Ge/Yb implies a sharper, less damped response, whereas the broader Ge/Y peaked points to a wider distribution of relaxation times and stronger damping. The amplitudes *a*_1_, *a*_2_, and *A* further suggest that Y and especially Yb alter the balance between localized-state relaxation and resonant transport and polarization. The stronger peak amplitude for Ge/Yb indicates that Yb enhances the high-frequency interfacial response more strongly than Y, which is consistent with a larger perturbation of the Ge electronic environment. Overall, the fitted parameters support the view that Y and Yb nanosheets create different densities and distributions of localized interfacial states, thereby changing carrier trapping, release, and polarization dynamics across the measured frequency range [28]. As also seen from the table, metal coating changes the interfacial carrier dynamics of Ge rather than only its conductance magnitude. Bare Ge and Ge/Y have a positive baseline term (*c*), while Ge/Yb shows negative (*c*), which suggests that the coated interfaces introduce a delayed or compensating current component associated with interface states, polarization, or trap-controlled charge exchange in the AC response. In admittance spectroscopy, such behavior is commonly linked to interfacial relaxation processes and non-ideal distributed responses rather than a simple ohmic channel [28,29].

It should be mentioned here that the fitting procedure was carried out using a high convergence minimization program based on the *χ*^2^ criterion. The uncertainty in the data was estimated to lie in the range of 2% to 10%. A representative best-fit result for the experimental data is shown by pink colored circles in Figure 4. The coefficient of residual sums is listed in Table 2.

### 3.4. Complex-Impedance (Nyquist) Analysis

**Figure 5:**
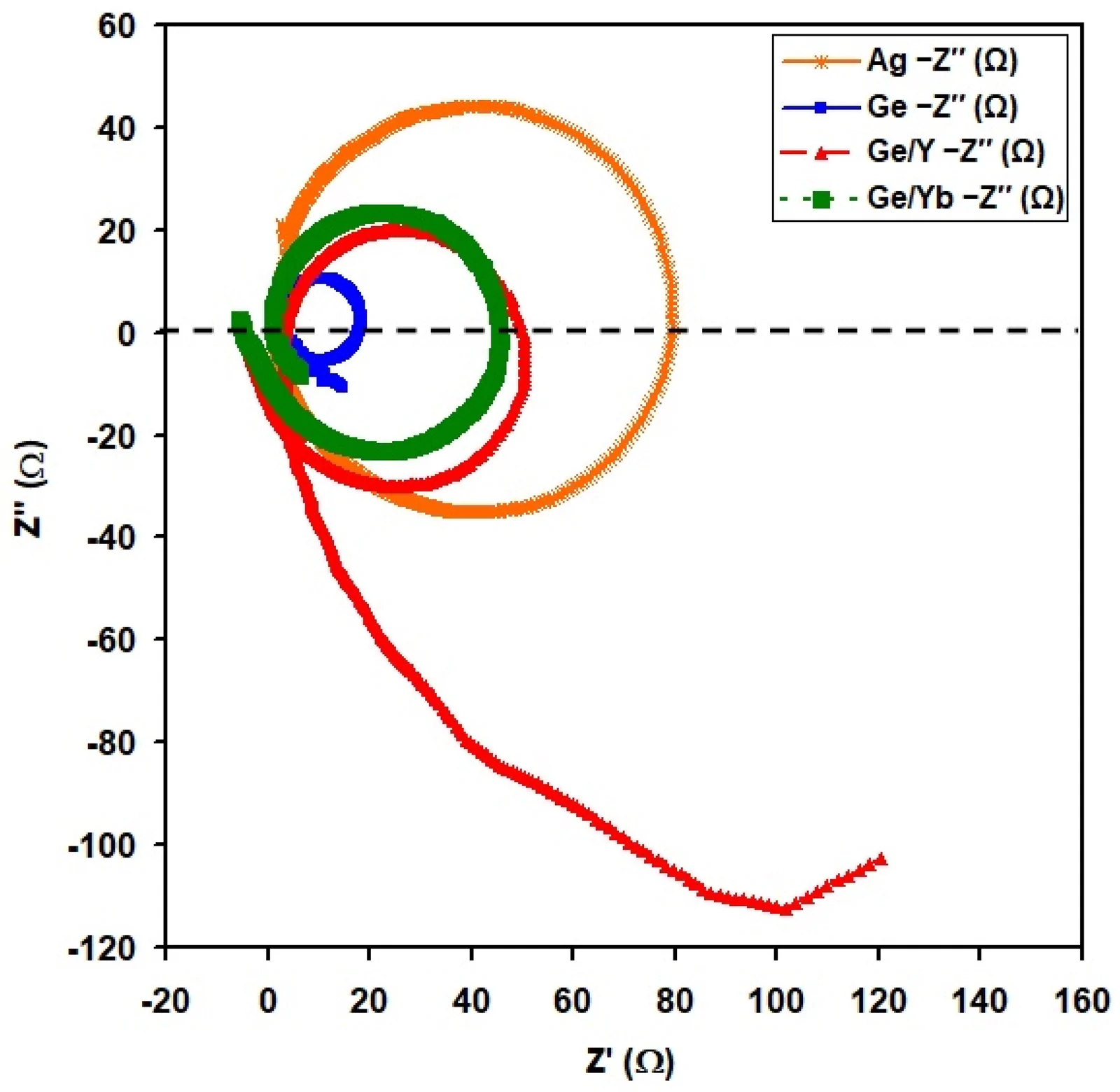
Nyquist diagrams *Z*^″^ versus *Z*^′^ for glass/Ag reference electrode and Ge, Ge/Y, and Ge/Yb structures, showing the effect of Ge film deposition and rare-earth Y and Yb films on complex impedance behavior.

The complex-impedance (Nyquist) diagrams for glass/Ag, Ge, Ge/Y, and Ge/Yb are presented in Figure 5. As seen from the figure, the glass/Ag control sample features a rather significant loop-like behavior with both positive and negative values of (*Z*^″^; imaginary part of impedance (*Z* = *Z*^′^ + *iZ*^″^)), which is clear evidence of the contribution of the lower Ag film to the frequency dependence of the reactive properties of the whole structures. High impedance of the glass/Ag control cannot be linked to the bulk resistivity of Ag, which is very good conductor of electricity, because the high impedance can only result from the frequency-dependent behavior of the electrode or interface. The deposition of Ge significantly reduces and changes the impedance curve, while the following layers of Y and Yb form completely different broad curves. Most notably, the Ge/Y structure exhibits a pronounced long tail extending toward high *Z*^′^ and strongly negative *Z*^″^, in addition to its loop-like region. This deviation from a simple semicircular response indicates an additional slow and broadly distributed relaxation contribution, consistent with enhanced interfacial polarization and trap-mediated charge redistribution in the Ge/Y structure. In contrast, Ge/Yb exhibits a more compact distorted loop, indicating a different distribution of relaxation processes. Overall, the non-ideal loops and particularly the extended Ge/Y tail demonstrate that a single ideal *RC* relaxation cannot describe these structures and support the presence of multiple, interface-dependent relaxation processes. The differences from the glass/Ag control further show that, although the Ag layer contributes to the reactive response, the observed behavior is determined by the collective response of the Ag electrode, Ge layer, and rare-earth-modified interfaces. In particular, the Ge/Ag and rare-earth-modified interfaces can introduce localized states and trapping centers that promote charge accumulation and interfacial polarization under the AC field. The Y and Yb layers modify the density and distribution of these interfacial states, leading to different charge-transfer and relaxation dynamics and, consequently, to the distinct Nyquist responses observed for Ge/Y and Ge/Yb.

### 3.5. Grounded Two-Port Microwave Thin-Film Network

**Figure 6:**
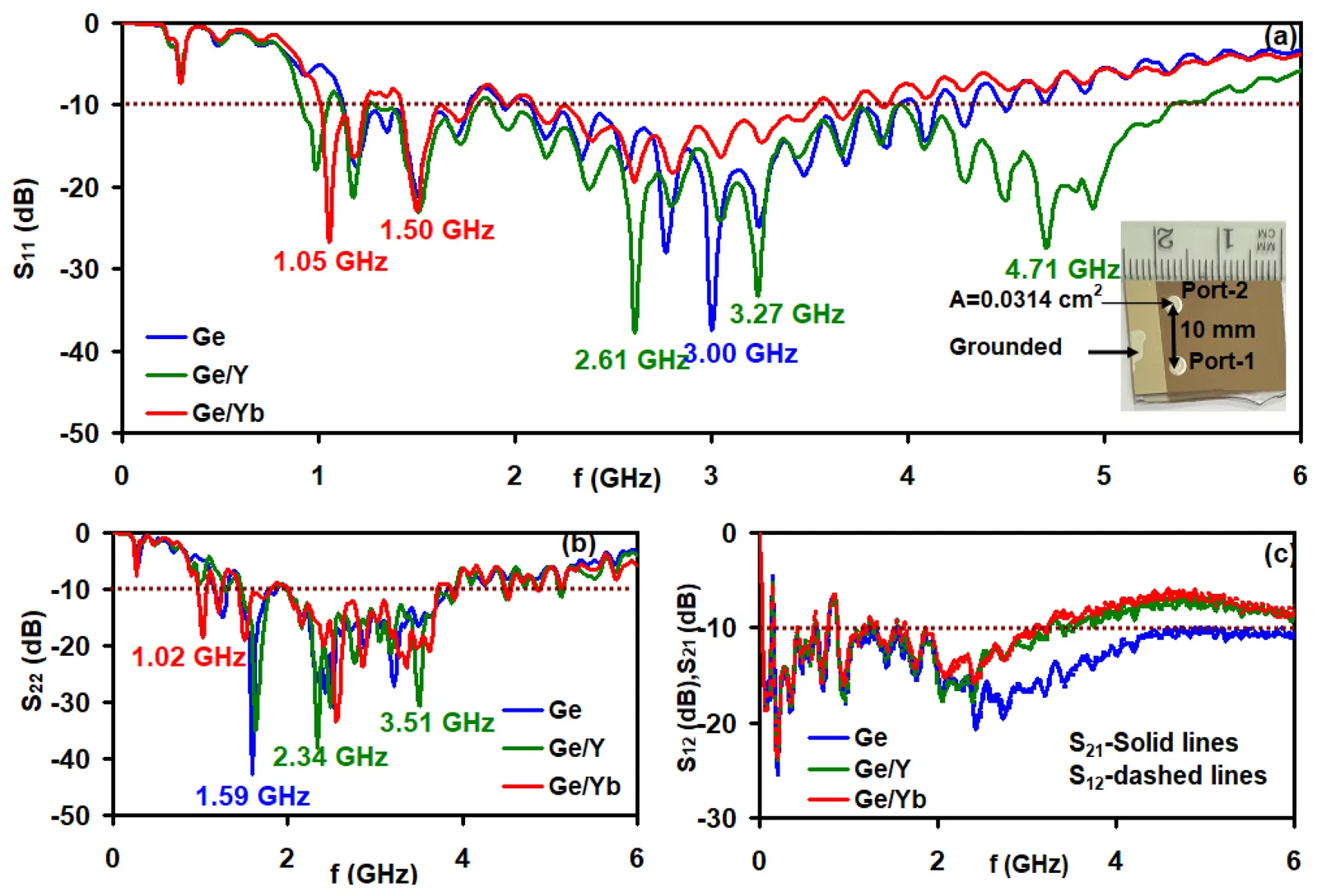
Measured scattering parameters of the Ge, Ge/Y, and Ge/Yb grounded two-port microwave thin-film networks: (**a**) port-1 reflection coefficient *S*_11_, (**b**) port-2 reflection coefficient *S*_22_, and (**c**) forward and reverse transmission coefficients *S*_12_ and *S*_21_. The inset in (**a**) illustrates the experimental two-port geometry, including the circular top contacts, their separation, and the common Ag ground. Dotted lines in the figures show the acceptable limits for these devices.

To analyze how modifications in electrical properties due to the presence of coating affect microwave-frequency performance of the structures under study, each sample was modeled as a grounded two-port microwave thin-film network. The measurement setup is schematically shown in the inset of Figure 6a. The samples included two identical circular Ag top electrodes of 0.0314 cm^2^ in size, separated from each other by 10 mm. The bottom Ag film was grounded via the common ground of the vector network analyzer. The two circular films formed ports 1 and 2, and the entire scattering matrix (including (*S*_11_, *S*_22_, *S*_21_, *S*_12_) was measured in the range of 0.01–6.0 GHz. The two ports were not simultaneously excited. For the measurement of *S*_21_, Port 1 served as the excitation/input port and Port 2 as the output port, while their roles were reversed for the measurement of *S*_12_. Accordingly, *S*_21_ and *S*_12_ represent the forward and reverse transmission/coupling coefficients of the grounded two-port structure, respectively. They are therefore interpreted here as measures of inter-port coupling in a microwave network rather than as radiation coupling between two antenna elements. In this case, *S*_11_ and *S*_22_ correspond to reflections at ports 1 and 2, while *S*_21_ and *S*_12_ are the transmissions between the ports. As no data on radiation properties of the samples was available, we will consider them as microwave two-port networks and not antennas.

The experimental two-port measurement geometry is illustrated in the inset of Figure 6a. Figure 6 itself compares the measured scattering parameters of the three grounded two-port Ge-based microwave network devices. The measurements were carried out in the spectral range of 0.01–6.00 GHz. In all cases, the device architecture consisted of two identical circular top contacts of area of 0.0314 cm^2^, separated by 10 mm, with the Ag back film connected to the common ground of the vector network analyzer. The signal terminal was applied sequentially to port 1 and port 2 to extract the complete two-port scattering matrix. This system is accepted as two-port microwave network (TPMN). The common Ag backplane provides the return path, while the two top contacts interact through the Ge-based surface layer and shared ground-supported current distribution [30].

The relationship between the electrical properties and microwave scattering characteristics of the investigated structures can be examined through a comparative experimental approach. Since the nominal device geometry and measurement configuration were maintained constant for Ge, Ge/Y, and Ge/Yb, the observed differences in their electrical and microwave responses are associated with the introduction of the rare-earth coatings. The temperature-dependent conductivity, frequency-dependent conductance and capacitance, and Nyquist impedance analyses demonstrate that Y and Yb coatings modify the electrical transport and interfacial relaxation characteristics of the Ge-based structures. Correspondingly, the measured scattering parameters exhibit coating-dependent variations in resonance frequencies, reflection coefficients, transmission coefficients, and impedance-matched frequency ranges. These observations establish an experimental correlation between the coating-dependent electrical properties and microwave network responses. Changes in the effective complex impedance, interfacial polarization, and frequency-dependent capacitive loading may contribute to the observed microwave behavior. However, the present experimental measurements do not allow the individual contributions of these mechanisms to be quantitatively identified. In particular, a definitive explanation of the measured S-parameter variations would require full-wave electromagnetic simulations incorporating experimentally determined frequency-dependent complex permittivity and conductivity, together with appropriate modeling of the electrode geometry, substrate, and measurement configuration. Furthermore, although identical nominal geometrical conditions and measurement procedures were employed, possible contributions from the measurement fixture cannot be completely excluded without electromagnetic de-embedding. Therefore, the proposed physical interpretation should be regarded as a plausible explanation consistent with the experimental observations rather than as proof of a specific electromagnetic mechanism.

It is clear from Figure 6a that the *S*_11_ (from port-1) spectra assures that all three devices reveal distinct resonant behavior. The resonance critical frequency values and the depth of the resonance vary based on the sample nature or surface treatment. As-grown Ge films exhibited absolute minima at 3.00 GHz, whilst yttrium-coated Ge shifted this absolute minima to 2.61 GHz. Yb-coated Ge showed absolute minima at 1.05 GHz. However, in TPMN design the bandwidth of the matched region is the practical important result rather than absolute minima. The scattering characteristics of port-2 (*S*_22_) are shown in Figure 6b. Considering the combined *S*_11_ and *S*_22_ behavior for each studied device, it is possible to say that yttrium coating show the broadest simultaneous matched band, covering the region of 2.01–3.90 GHz, compared with 2.10–3.81 GHz for uncoated Ge and 2.10–3.51 GHz for the Yb-coated structure. It means that Y nanosheet coating is the more effective of the two rare-earth modifications for broadening the useful shared operating range. It can also be seen from Figure 6b that *S*_22_ spectra reveals the same conclusions. Although the Ge, Ge/Y and Ge/Yb TPMN show the absolute minima at 1.59 GHz, 2.34 GHz and 2.55 GHz, respectively, the Ge/Y device shows the most balanced two-port behavior in the main microwave operating band. At the best common operating point near 2.40 GHz, the Y-coated sample displays *S*_11_ = −19.74 dB and *S*_22_= −20.08 dB, which is more symmetric than the Yb-coated sample and slightly more balanced than the uncoated Ge device. The improved balance between the two-port reflection coefficients following Y coating is experimentally observed and may be associated with changes in the effective electrical and interfacial properties of the structure. However, the precise contribution of surface impedance, capacitive loading, and electromagnetic field redistribution cannot be established from the measured scattering parameters alone and requires further electromagnetic modeling. Yttrium-oxide-based thin films are mentioned exhibiting high-(*κ*) dielectric materials whose electrical response depends strongly on microstructure and defect content, which makes such an interpretation physically reasonable [31].

As seen in Figure 6c, the forward (*S*_21_) and the reverse (*S*_12_) transmission coefficients of the two ports of the studied microwave devices are presented. From the nearly identical behavior of *S*_21_ and *S*_12_, the two-port system is reciprocal for almost the entire frequency band being examined. The values of these transmission coefficients, however, are not equal for Ge, Ge/Y, and Ge/Yb samples. Particularly, the uncoated Ge structure exhibits the strongest sustained inter-port decoupling, with both *S*_21_ and *S*_12_ remaining below −15 dB from 2.01 to 3.24 GHz. In Y-coated films, the common (*S*_12_ and *S*_21_ > 15) dB isolation region narrows to 2.01–2.43 GHz, although the wider (*S*_12_ and *S*_21_ > 10) dB isolation band remains broad, extending from 1.56 to 3.24 GHz. In contrast, the Yb-coated sample exhibited the weakest “low inter-port transmission”, with (*S*_12_, *S*_21_ < −15) dB only in a very narrow region of 2.40–2.43 GHz. As a result, the coatings do not improve all figures of merit simultaneously: Y coating enhances the continuity and width of the common matched band, whereas the uncoated Ge sample still gives the best extended high-isolation performance, and Yb coating is the least favorable for port decoupling. The different reflection and transmission characteristics observed for Ge, Ge/Y, and Ge/Yb indicate that rare-earth surface modification influences the overall microwave network response. The electrical measurements suggest that coating-dependent changes in carrier localization, interfacial polarization, and relaxation processes may contribute to these differences. Previous studies have also reported that the dielectric properties of yttrium- and ytterbium-based materials are sensitive to their microstructure and interfacial conditions [7,31]. Nevertheless, these considerations provide only a possible physical interpretation of the observed scattering characteristics. Without experimentally determined complex dielectric parameters and full-wave electromagnetic modeling, the relative contributions of material-dependent impedance changes, capacitive loading, and geometrical electromagnetic effects cannot be quantitatively resolved.

Considering the measurement results of the two-port network responses, all three TPMNs have ranges where *S*_11_ and *S*_22_ < −10 dB [32], implying low reflection levels at both ports within some part of the tested sub-6 GHz frequency band. Furthermore, the transmission parameters *S*_21_ and *S*_12_ demonstrate significant dissimilarity in the inter-port coupling of the three TPMNs. This describes the microwave matching and coupling properties of the current thin-film two-port networks.

From a technological perspective, the most important observation is that the three investigated grounded two-port microwave thin-film networks exhibit distinct reflection and transmission characteristics within the 2–4 GHz frequency range, which overlaps with frequency bands relevant to sub-6 GHz wireless communication systems, including 5G NR Frequency Range 1 (FR1) [33]. These findings indicate that the investigated structures may provide a basis for future development of microwave components operating within these frequency bands. However, the present measurements characterize only the scattering parameters, impedance matching, and inter-port coupling of the grounded two-port networks. Since radiation patterns, antenna gain, and radiation efficiency were not measured, the structures cannot currently be classified as validated antennas or MIMO antenna systems. Further electromagnetic simulations and experimental radiation measurements would be necessary to establish their suitability for antenna applications. Within the scope of the present investigation, Y coating provides the broadest common impedance-matched frequency range, whereas uncoated Ge exhibits the strongest sustained inter-port isolation. Thus, rare-earth surface modification offers a promising approach for tailoring the microwave network response of Ge-based thin-film structures.

For the benefit of the reader, Table 3 provides a summary of the key results for all three Ge-based two-port microwave networks coated with different nanosheets and allows for a comparison to be made regarding the impact of Y and Yb nanosheet coating. From the results, it is evident that the sample coated with Y nanosheets possesses the broadest impedance-matched frequency range, covering 2.01–3.90 GHz, while the uncoated Ge sample possesses the strongest sustained high-isolation characteristics, achieving *S*_12_, *S*_21_ < −15 dB over 2.01–3.24 GHz. However, it is also evident that the sample coated with Yb nanosheets possesses the weakest high-isolation region, highlighting a weak decoupling characteristic. From this table, it is evident that Y nanosheet coating is the best method for improving the operating bandwidth, while the uncoated sample is again superior for achieving strong inter-port isolation.

Future investigations should focus on determining the frequency-dependent complex permittivity and conductivity of the Ge, Ge/Y, and Ge/Yb structures and incorporating these parameters into full-wave electromagnetic simulations. Such analyses would enable the separate evaluation of geometrical and material contributions to the measured microwave response and facilitate optimization of the contact dimensions, port separation, and layer thicknesses. Although the present structures exhibit promising microwave network characteristics, their potential application as radiating antenna devices remains to be established through additional electromagnetic simulations and experimental measurements of radiation patterns, gain, and radiation efficiency.

## 4. Conclusions

In the current study we have employed the rare-earth metal coating technique to enhance the performance of amorphous germanium thin films. Two metal, Y and Yb, nanosheets of thickness of 100 nm were coated onto the surface of 500 nm thick germanium thin film. The films were structurally, morphologically and electrically characterized. Although the metal nanosheets coating did not alter the amorphous nature of the films, the temperature-dependent electrical conductivity analyses which was held in the temperature range of 20–300 K showed enhanced electrical conductivity values and stabilized temperature independent conductivity, especially in the temperature range of 20–120 K. It was also observed that the deposited metal nanosheets significantly modify the localized-state distribution and carrier transport pathways in the films. In addition, impedance spectroscopy analyses in the frequency domain of 0.001–1.80 GHz have shown that Ge thin films are sources of negative capacitance effect and positive conductance, whilst Y- and Yb-coated films exhibited both negative capacitance and negative conductance effect. Furthermore, all three Ge-based fabricated grounded two-port structures possessed different microwave reflection and transmission behavior in the range of 0.01–6 GHz, thus illustrating that rare-earth element surface treatment is an effective approach to tuning the microwave network behavior of the Ge thin films. In this regard, the broadest low-reflection range for all three structures was achieved with Y, while the uncoated Ge thin film possessed the highest decoupling between ports. Therefore, it can be concluded that rare-earth surface treatment provides an effective approach for tailoring the electrical and microwave network characteristics of Ge-based thin-film structures. The present findings demonstrate their potential for further development as microwave components operating in the sub-6 GHz frequency range. However, the investigated structures have been characterized exclusively as grounded two-port microwave networks, and no antenna radiation properties have been evaluated. Consequently, their possible application as radiating devices requires further investigation through full-wave electromagnetic simulations and experimental characterization of radiation patterns, gain, and radiation efficiency.

## Figures and Tables

**Table 1:** VRH parameters for uncoated and metal-coated Ge thin films.

Sample	Range (K)	*T_o_*(×10^6^ K)	N(E_F_) cm^3^/eV	W (meV)	R (Å)	GR
Ge	200–290	483.7	4.34 ×10^17^	201.09	139.89	13.99
Ge/Y	200–290	559.8	3.75 ×10^17^	208.58	145.10	14.51
Ge/Yb	200–290	669.4	3.14 ×10^17^	218.11	151.73	15.17
Ge	115–200	49.08	4.28 ×10^18^	81.21	88.27	8.83
Ge/Y	145–200	2.66	7.88 ×10^19^	39.20	42.61	4.26
Ge/Yb	160–200	1.036	2.03 ×10^20^	30.95	33.64	3.36

**Table 2:** Fitting parameters of the frequency-dependent conductance spectra for the Ge, Ge/Y, and Ge/Yb channels obtained using the extended frequency-domain models.

Parameter	Ge	Ge/Y	Ge/Yb
c (Ω^−1^)	0.29	0.50	−0.21
*a*_1_ (Ω^−1^)	0.31	−0.73	0.06
*a*_2_ (Ω^−1^)	−0.26	−0.45	0.24
A (Ω^−1^)	0.29	0.31	0.74
*f_o_* (GHz)	1.54	1.33	1.65
*f*_1_ (GHz)	0.28	0.89	0.01
*f*_2_ (GHz)	0.53	1.11	0.69
*τ_o_* (ns)	0.10	0.12	0.10
*τ*_1_ (ns)	0.57	0.18	28.08
*τ*_2_ (ns)	0.30	0.14	0.23
γ (GHz)	0.06	0.05	0.02
m_1_	2.76	2.61	3.35
m_2_	5.03	4.25	7.18
R^2^	0.9982	0.9990	0.9964

**Table 3:** Comparison of scattering parameters, impedance-matched bands, and inter-port isolation characteristics of Ge, Ge/Y, and Ge/Yb microwave networks.

Sample	*S*_11_ (dB)	*S*_22_ (dB)	*S*_21_ @0.21 GHz (dB) *	*S*_12_ @0.21 GHz (dB) *	Common Matched Band (GHz) *S*_11_, *S*_22_ < −10 dB	Common Isolation Band (GHz) *S*_21_, *S*_12_ < 10 dB	Balanced Operating Frequency (GHz)
Ge	−37.42 dB at 3.00 GHz	−42.75 @ 1.59 GHz	−25.56	−25.22	2.10–3.81	2.01–3.24	2.73
Ge/Y	−37.84 @ 2.61 GHz	−38.06 @ 2.34 GHz	−24.12	−23.96	2.01–3.90	1.56–3.24	2.40
Ge/Yb	−26.71 @ 1.05 GHz	−33.24 @ 2.55 GHz	−23.82	−23.57	2.10–3.51	1.65–3.06	2.40

* Note: The *S*_21_ and *S*_12_ values at 0.21 GHz represent the low-frequency transmission characteristics shown in Figure 6c. The common matched bands correspond to *S*_11_ and *S*_22_ < −10 dB, whereas the common isolation bands indicate frequency ranges of reduced inter-port transmission, as discussed in Section 3.5.

## Data Availability

The data that support the findings of this study are available from the corresponding author upon reasonable request.
